# Selective Particle Filtering in a Large Acoustophoretic Serpentine Channel

**DOI:** 10.1038/s41598-019-43711-8

**Published:** 2019-05-09

**Authors:** M. H. Kandemir, R. M. Wagterveld, D. R. Yntema, K. J. Keesman

**Affiliations:** 1 Wetsus, European Center of Excellence for Sustainable Water Technology, Oostergoweg 9, 8911 MA Leeuwarden, The Netherlands; 2 Biobased Chemistry & Technology/Mathematical and Statistical Methods (Biometris), Wageningen University & Research, Bornse Weilanden 9, 6708WG Wageningen, The Netherlands

**Keywords:** Mechanical engineering, Environmental biotechnology

## Abstract

The objective of this study is to investigate the performance of a serpentine channel for acoustically driven selective particle filtering. The channel consists of sharp corners and straight sections, and the acoustic field is affecting the particles throughout the channel. A prototype of the separator channel is manufactured using 3D printing. Acoustic waves are generated by a piezoelectric transducer operating near 2 MHz. Computer simulations are carried out to explore and visualize the flow field and acoustic field in the separator. Selective particle trapping is aimed to be achieved in the hairpin sections, which is confirmed by experiments. Spherical polyethylene particles of 34 µm, 70 µm and 100 µm diameter are used to demonstrate selective trapping by adjusting the flow rate in the channel or voltage input to the transducer. In addition, wheat beer containing yeast up to 20 µm size is selectively filtered by adjusting the flow rate to the channel. Experiments demonstrate that selective particle filtering is possible in the serpentine channel as both methods yield clear separation thresholds.

## Introduction

Manipulation of particles by acoustics is known as “acoustophoresis”. Applications of acoustophoresis cover a wide range of applications, from centimeter-scale^1^ to micrometer-scale sized particles. In the micrometer-scale, acoustophoresis is one of the methods for particle filtering in solid-liquid mixtures among inertial, gravitational, magnetic and optical methods^2–6^.

Enhanced sedimentation is one of the common applications of acoustophoresis in particle-liquid separation^7–9^. Particles are aggregated by virtue of the acoustic radiation force and sedimentation is thus enhanced. This method can be combined with flow fields to selectively retain viable cells^10–13^. Apart from enhanced sedimentation, most applications in acoustophoresis utilize a single acoustic node in the separator channel. Since the frequency is often high and the wavelength is therefore low, dimensions of such channels are in micrometer range. Hence, these are referred to as microchannels. Selective separation for model particles and bioparticles using microchannels are demonstrated in a number of studies^14–18^. Selective particle retention and fractionation can also be achieved with methods other than enhanced sedimentation and acoustophoresis. Examples are inclined sedimentation^19^, dielectrophoresis^20–22^, and flow manipulation^23–25^. Acoustic filtering can be an alternative for existing applications in food processing^6^. For example, in rough beer filtration processes, membranes recently offer a competitive alternative to traditional kieselguhr method^26–28^. The potential of acoustic filtration is yet to be explored for this application. Possible benefits include the absence of fouling, no addition of chemicals, not having internals and less cleaning requirement.

A disadvantage of a single node channel is the relatively low throughput. The throughput can be increased by operating multiple set-ups in parallel^29^, but also by employing multiple nodes. To have multiple nodes in the resonator, the resonator dimensions must be around multiple wavelengths at the excitation frequency. In such systems, it is also possible to employ relative motion between the fluid flow and the acoustic field, by either pushing the flow against the acoustic field or creating a dynamic acoustic field^30^. Recently, multi-wavelength setups utilizing surface acoustic waves have been proposed^31^. A review on multiple wavelength resonators was done by Hawkes *et al*.^32^.

To understand acoustic particle filtering, it is crucial to know how the acoustic field affects particles. A body in an acoustic field experiences a force called acoustic radiation force. Expressions for the acoustic radiation forces on spherical particles were presented by King, for traveling and standing wave fields^33^. Later, an expression for compressible particles in a generic acoustic field was developed by Gor’kov^34^. As compared to traveling wave fields standing wave acoustic fields have higher energy density, thus leading to higher time-averaged forces^33^. In acoustic standing wave separators particles are trapped in specific grid points (the acoustic nodes, or antinodes), thus making it possible to separate particles from the suspension^6^.

Depending on the particle properties, acoustically different particles can be forced to different locations in the same acoustic field. When a particle moves relative to the liquid, the particle will also experience a so-called drag force. If the liquid flow is characterized by a low Reynolds number, the drag force is typically modeled as Stokes drag force^35^. Interestingly, some particles are trapped with relatively lower effort compared to others as acoustic radiation force scales with particle properties such as volume, density and compressibility while drag force scales with particle size. Such diverse behavior can be used in selective filtering using acoustic fields.

Combining those external forces on particles, the equation of motion for a particle can be constructed, which is a second-order nonlinear ordinary differential equation. In general, particle trajectories can be calculated numerically, but under some assumptions analytical solutions are also possible. The solution of the equation of motion can be combined with the flow profile in the separator, leading to more complex modeling^36–40^. Thus, the dominant external forces on a particle in an acoustic field are drag force and acoustic radiation force. Being able to adjust those external forces enables the particles to be selectively trapped. If the particles are pushed relative to the acoustic field by the fluid flow, the drag force can be controlled by the fluid velocity whereas the acoustic force can be controlled by the voltage input to the system. A serpentine channel consisting of hairpins and linear sections can be used to control the fluid flow and acoustic input.

The aim of this study is to investigate the possibility for selective filtering in an acoustophoretic serpentine channel and to demonstrate it on solid polyethylene particles and on wheat beer flocs. The width of the channel is such that multiple wavelengths at operating frequencies fit in the channel. Furthermore, the channel dimensions and shape are chosen such that particles are trapped in multiple nodal locations, while keeping the liquid flow inside the channel close to laminar. The serpentine structure has low velocity hairpin turns connected by straight sections. Drag force on the particles becomes weaker in the hairpins and the straight sections ensure that the flow becomes fully developed before entering the hairpins. This selective filtering ability is shown in computer simulations and in experiments using a 3D printed prototype, by adjusting the flow rate in the channel or voltage input to the system. Model polyethylene particles of 34 µm, 70 µm and 100 µm diameter are used to demonstrate the ability of selective particle separation in the prototype by either changing the input flow rate or voltage input to the transducer. The other test mixture, wheat beer, contains yeast up to 20 µm in size and has a more or less continuous size distribution of yeast particles. Wheat beer is used to demonstrate the ability to selectively separate compressible flocs by changing the input flow rate.

## Materials and Methods

**Figure 1:**
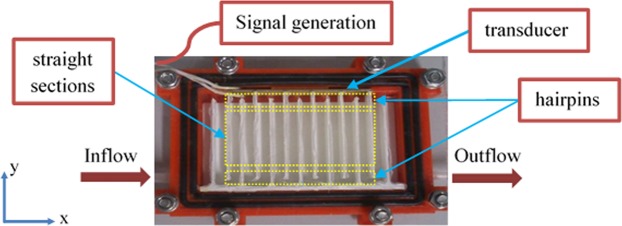
Schematic top view representation of the system. Red part in the separator is the base, white parts are inner walls. Black parts are rubber rings. Yellow rectangles highlight the hairpins and the straight sections among the inner walls. To ensure the flow is following the serpentine path, 1-mm thick silicone sealant layer is also applied between the inner walls and the PMMA cover.

The experimental set-up consists of a signal generator (Keysight Trueform 33512B), a custom-made amplifier, an oscilloscope (Tektronix TDS2024C), a syringe pump (Aitecs PRO SP-12S) and the separator prototype, Fig. 1. The amplifier drives the piezoelectric transducer (Noliac NCE41), the oscilloscope monitors the voltage across the transducer. While acting as a sound source, the piezoelectric transducer is immersed in water and in order to prevent electrical leakage it was covered with polyurethane paint. The particle-water mixture is fed by the syringe pump. Base and inner wall parts of the separator prototype are 3D printed from Polylactic acid (PLA) using Ultimaker 2 +. Base part contains the inlet and outlet sections, connection holes for the top PMMA cover and beds for rubber rings. Inner part creates the serpentine structure inside by having straight sections and 180 ° turns, i.e. hairpins. During the experiments the separator was always laid on the horizontal plane.

Two particle mixtures were used in the experiments. Mixture (1) contains model spherical polyethylene particles with known dimensions and density. Mixture (2) is a commercially available wheat beer.

The properties of particles of mixture (1) are listed in Table 1. A bottle of mixture consists of 500 mL MilliQ water, 0.84 g L^−1^ of the surfactant CTAB (Hexadecyltrimethylammonium bromide), 0.99 mM HCl and 0.02 g L^−1^ of each particle type, while multiple bottles of mixtures are consumed during the experiments. CTAB prevents the particles from aggregating and the PMMA cover whereas HCl prevents the particles from sticking to the PLA parts. Mixture (2), Hoegaarden wheat beer, contains dissolved carbon dioxide in very small bubbles and it produces foam when moved or shaken. To be able to remove all carbon dioxide the beer mixture was acidified. The acidification shifts the carbonate equilibrium towards carbonic acid and dissolved carbon dioxide. The gas was removed by keeping the solution under vacuum for 2.5 hours.

**Table 1:** Properties of the polyethylene particles in the mixture.

Color	Blue	Orange	Red
Size (*μm*)	90–106	63–75	32–36
Density (*kgm*^−3^)	1002	1006	980
Speed of Sound (*ms*^−1^)	1720	1717	1739

During the treatment of mixture (1), the selective separation thresholds were determined visually using a microscope with camera and ImageJ^41^. Whether particles got trapped or not was first checked by observing the experiments live and later verified by analyzing the particle trajectories using ImageJ. Separation thresholds were adjusted by changing the voltage input or flow rate for mixture (1). Prior to operation, the admittance of the system, filled with degassed MilliQ water and particle mixtures, was measured with an impedance analyzer (HP 4194 A) between 1800–2000 kHz. Computer simulations were carried out to compute the acoustic field, where a frequency of 1979 kHz was selected as excitation frequency, since at 1979 kHz the acoustic field appeared to be the most favorable for trapping in the hairpins. Admittance calculations by computer simulation, with an isotropic structural loss factor of 0.1 included in the PLA material, suggested that there is more than one resonance in the frequency range of 1800–2000 kHz. The selected frequency of 1979 kHz coincided with the frequency of minimum imaginary admittance in the simulations. In the measurements the phase is closest to zero at 1924 kHz in mixture (1) and 1972 kHz in mixture (2) indicating the minimum imaginary admittance occurs there. Thus, excitation frequencies for both mixtures are chosen accordingly. For mixture (2), separation threshold was adjusted only by changing the flow rate. After separation, the particle size distributions were analyzed with a DIPA 2000 particle size analyzer.

The dimensions of the acoustic separator allow for multiple wavelengths in the operating frequency range. Ideally, in this type of resonator the acoustic field is a one-dimensional standing acoustic plane wave field. The equation of motion for a spherical particle in such a field is given by

1 $$(\frac{4}{3}\pi {r}^{3}\rho )\ddot{y}+(6\pi \mu r)(\dot{y}-u)+4\pi k{r}^{3}(\frac{{P}_{0}^{2}}{4{\rho }_{0}{c}_{0}^{2}}){\rm{\Phi }}(\rho ,c)\sin (2ky)=0$$

Here *r* is the particle radius (m), *y* is the distance of particle from the source (m), *ρ*_0_ is the density of the medium (kg m^−3^), *c*_0_ is the speed of sound (m s^−1^) in the medium, *P*_0_ is the amplitude of the sound pressure (Pa), ω is the angular frequency of excitation (rad s^−1^), *k* is the wavenumber (m^−1^) and Φ(*ρ,c*) is the acoustic contrast factor. The acoustic contrast factor is a measure on how much the particle differs from the surrounding media in terms of acoustics^36^. Particle density is denoted by ρ whereas c represents the speed of sound in the particle, *μ* is the dynamic viscosity (Pa s) of the medium, *u* is the velocity (m s^−1^) of the flow in the direction of acoustic radiation force. With a known acoustic field and particle properties, Eq. (1) can be solved numerically in order to predict particle trajectories. Application of the acoustic radiation force on a particle is readily available in COMSOL Multiphysics.

**Figure 2:**
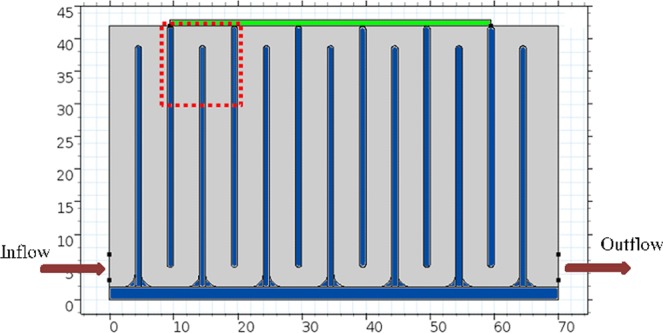
2D representation of the separator for computer modeling. Size of the transducer (green) on top of flow cell is 50 mm by 1 mm. In the flow cell the thickness of the inner walls (blue) is 1 mm and length of the walls is 37 mm. Channel width is 5 mm before the outlet and 4 mm elsewhere. Bottom corners are rounded with 1 mm radius. The area monitored during experiments is indicated by the red dotted rectangle.

The flow field and the acoustic field inside the separator were computed using COMSOL Multiphysics, version 5.3a. The 2D computer representation of the separator is given in Fig. 2.

The flow field is computed by a stationary solver in COMSOL Multiphysics, obtaining the steady state fluid velocity distribution in the separator. The mesh size is selected such that the maximum elements size is 3.71 mm, with an average element quality of 0.834 given by the software.

Acoustic field simulations of the separator were also carried out in COMSOL Multiphysics, using acoustic-solid interaction physics. Electrostatics, solid mechanics and pressure acoustics physics are used to calculate the acoustic field by frequency domain solver. Laminar flow physics is used to calculate the flow field by stationary solver. Particle paths are calculated using particle tracing for fluid flow physics by time dependent solvers. Multiphysics interactions and boundary/compatibility conditions are automatically generated by the software.

The transducer material is Noliac NCE41 and the dimensions are 1 mm × 10 mm × 50 mm. Piezoelectric properties of the transducer are adopted from previous work of Cappon^6^. Water properties are taken as built-in values in COMSOL Multiphysics. Properties of PLA are set as ${\rho }_{PLA}=1300\,kg{m}^{-3}$, ${E}_{PLA}=1.28\,GPa$, $\nu =0.36$ (Poisson’s ratio) and the isotropic structural loss factor of 0.1. Outer walls are represented as impedance boundary condition while inner walls are included in the simulations by including the PLA material. Simulations are carried out for frequencies between 1.8 MHz and 2.2 MHz with 500 Hz steps. Voltage input to transducers was set to 2 V_pp_. The mesh is adjusted so that the maximum element size is smaller than *λ*/8 for every simulated frequency in the corresponding material. There are 1458967 elements in total, where the average element quality is given as 0.9 by COMSOL Multiphysics. Boundary and compatibility conditions at material interfaces and multiphysics couplings are also generated by COMSOL Multiphysics. In addition to the acoustic field, the impedance and admittance of the transducer were also calculated. The resonant frequency of the system can also be found by the admittance plot due to the coupling between the system and the piezoelectric material^42^. Based on the computed flow and acoustic fields, particle trajectories were simulated.

## Results and Discussion

**Figure 3:**
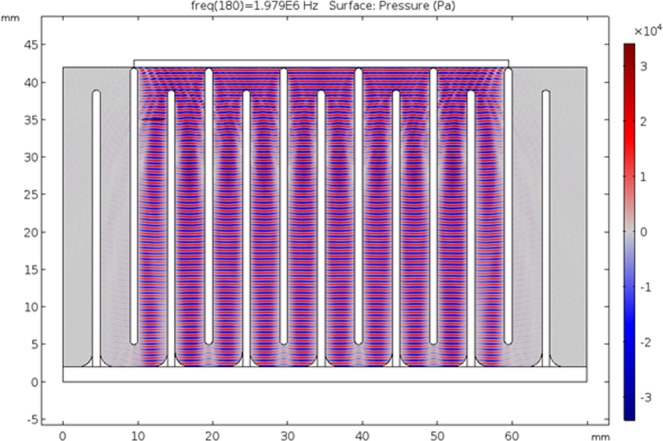
Acoustic pressure field (Pa) computation in COMSOL Multiphysics at 1979 kHz with 2 V_pp_ potential difference across the transducers. Red areas represent positive acoustic pressure where blue areas represent negative acoustic pressure. Grey lines between the blue and red areas corresponding to pressure nodes.

In order to gain insight of the properties in the system, first computer simulations were carried out to calculate the resonance frequencies, the acoustic field and the flow field. Excitation frequency of the system was selected based on admittance measurements and simulations. A simulation plot of the acoustic field corresponding to 1979 kHz is given in Fig. 3. Voltage across the transducers is set to 2 V_pp_ during the simulations. The acoustic field, more or less perpendicular to the flow direction, is strongest with the highest absolute sound pressure in the most colored (red and blue) area in Fig. 3.

**Figure 4:**
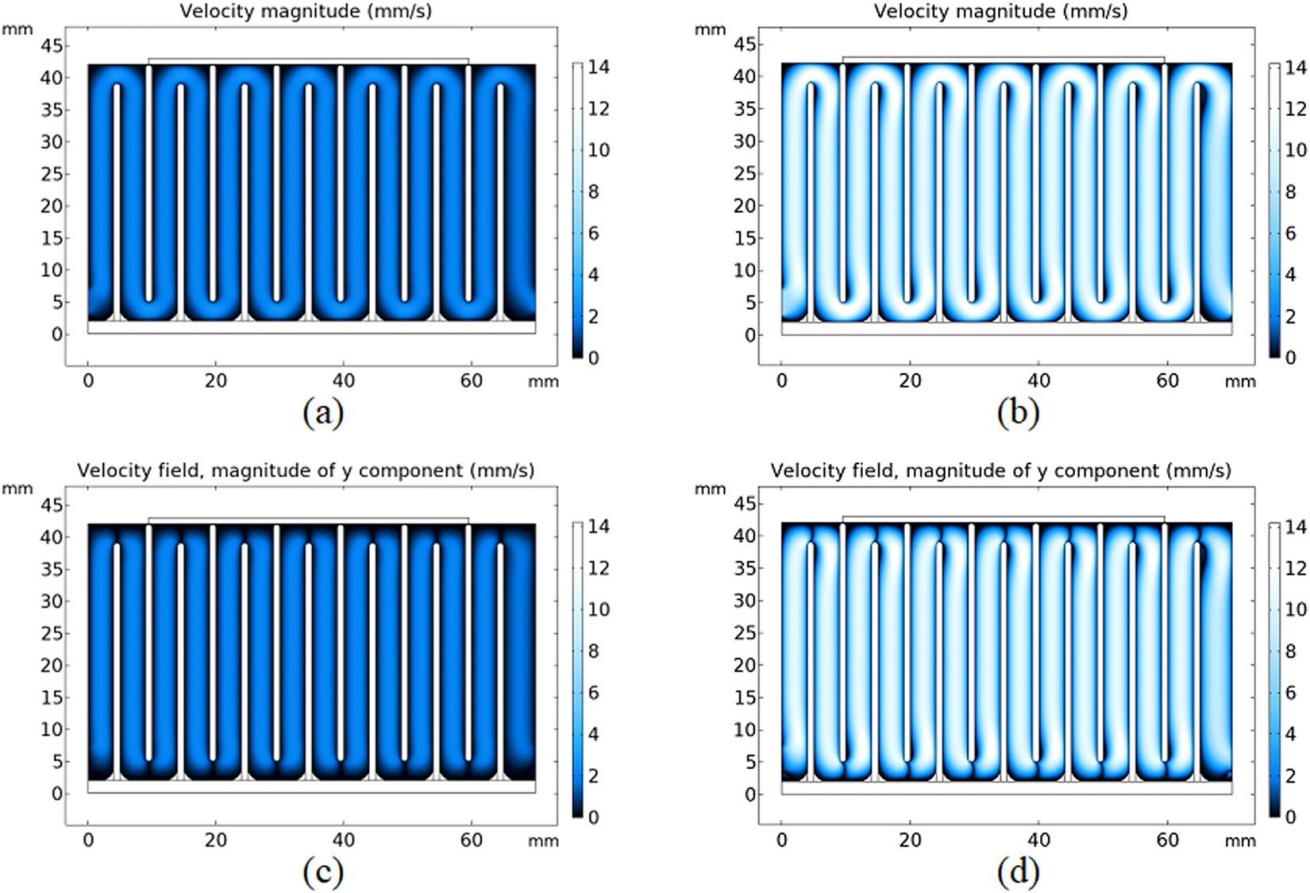
Flow field solutions in COMSOL Multiphysics. (**a**) Velocity magnitude (mm s^−1^) plot for 100 mL h^−1^ flow rate. (**b**) Velocity magnitude plot for 500 mL h^−1^ flow rate. (**c**) Magnitude plot of vertical velocity for 100 mL h^−1^ flow rate. (**d**) Magnitude plot of vertical velocity for 500 ml h^−1^ flow rate.

The flow field was computed in COMSOL Multiphysics and given in Fig. 4. The flow field solutions suggest that the flow is close to laminar even for the highest possible flow rate for this experiment. Figure 4(a) represents the flow inside the channel for an input flow rate of 100 mL h^−1^ and (b) represents the flow for an input flow rate of 500 mL h^−1^. For a flow rate of 100 mL h^−1^ Reynolds number is around 5. The channel provides a fully developed flow in the middle sections and for a flow rate of 500 mL h^−1^ the Reynolds number is around 25, indicating that the flow inside the channels is indeed close to laminar. Higher Reynolds numbers affect the flow especially around the corners, resulting in leaning as can be seen in Fig. 4(b). Figure 4(c,d) shows the vertical velocity amplitudes.

**Figure 5:**
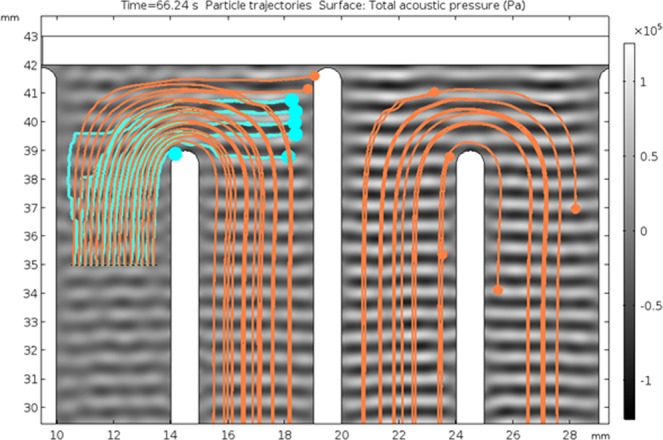
2D simulation of particle paths in COMSOL Multiphysics for 100 μm (blue) and 75 μm (orange) particles. The input flow rate is 100 mL h^−1^. Standing wave pattern is presented in grayscale.

Particle paths have also been simulated in COMSOL Multiphysics for the acoustic field (Fig. 3) assuming 100 mL h^−1^ flow rate (Fig. 4). Only the blue and orange particles were included in this simulation. Of the 15 particles released of each type to the system all of blue particles and 2 of orange particles are trapped in the first hairpin. The remaining orange particles continued unobstructed. The 2D simulations in Fig. 5 do not take into account the variation of the acoustic field along the depth of the prototype, which differs from the real experimental case as shown in what follows. Figure 5 also confirms that small particles are trapped, as well, by the acoustic field if they are located in the outermost streamline, where the drag force in vertical direction is the weakest.

The dominant external forces on a particle are drag force and acoustic force according to Eq. (1). Hence, by changing the voltage input, the amplitude of the sound pressure, and thus acoustic force, can be adjusted whereas it is possible to adjust the drag force by changing the input flow rate to the system. Mixture (1) is selectively filtered by changing the acoustic force or drag force; and mixture (2) is selectively filtered by changing the drag force only. Mixture (2) contained smaller particles with unknown properties. In this case, the maximum voltage input was used to ensure the particles are trapped and the flow rate was controlled to achieve selectivity.

**Figure 6:**
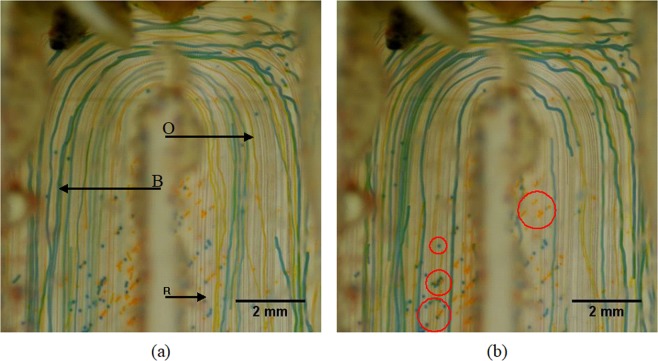
Particle path lines at the monitoring area of the prototype, recorded by stacking two sets of images over 30 s in 60fps. Blue (B), orange (O) and red (R) lines represent the path lines of the corresponding particles while the system is operated at 26% of the maximum voltage available and 100 mL h^−1^. The path lines are indicated by arrows in (**a**). Some particles that are stuck in the grooves of the 3D printed base are indicated by circles in (**b**).

To investigate the selective filtering capability by adjusting the voltage input, mixture (1) is pumped through the experimental flow cell with a constant flow rate of 100 mL h^−1^. For this flow rate it is possible to trap all particles by applying the maximum available voltage. When the acoustic field is off, no particle trapping is observed and the particle size distributions before and after passing through the cell were similar. Selective separation thresholds were determined visually while adjusting the voltage input to the transducers, and subsequently verified by analyzing the particle trajectories using ImageJ. Particles are trapped in the hairpins of the separator rather than in the straight sections. This behavior is due to the lower vertical flow velocities in the hairpin corners where the drag force is small enough to be overruled by the acoustic force. Figure 6 presents the particle path lines in the monitored area of the separator.

**Figure 7:**
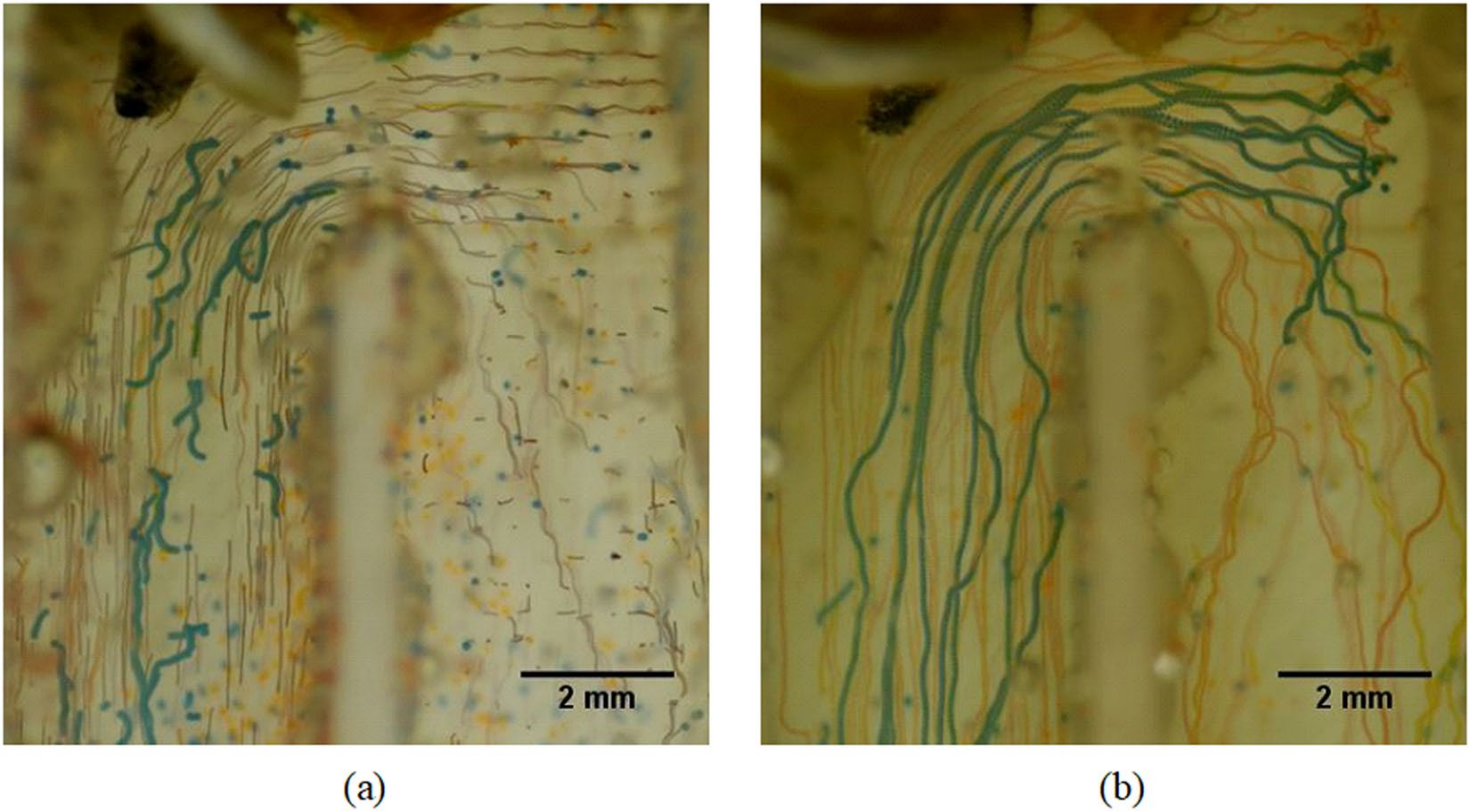
All blue, orange and red particles are trapped at 96% of the maximum available voltage input (**a**) and selective trapping of blue particles with 26% of the maximum available voltage input (**b**). In case (**b**), there are no red particles in the mixture. In both cases, flow rate was set to 100 mL h^−1^.

Blue and orange particles are affected by the acoustic field and red particles are continuously unaffected. The path lines of the red particles follow the streamlines of the laminar flow field, as can be seen in Fig. 6. The corresponding path lines of the blue, orange and red particles are indicated in the left panel of Fig. 6. There are also some stationary blue and orange particles in the pictures. Those are trapped by the acoustic field before recording and settled in the small surface grooves of the 3D printed base. As a result of imperfections in the acoustic field as well as variations in the third dimension, some particles escaped the trapping positions and continued with the flow. Even though some blue and orange particles initially continued with the flow, they were eventually trapped in one of the following hairpins. Selective filtering of particles is achieved by changing the voltage input. The maximum available voltage input is 50 V_pp_ across the transducer. When increasing the voltage first the largest particles (blue) were retained in the system with 26% of the maximum voltage, followed by the largest and medium (orange and blue) particles with 48% of the maximum voltage. Lastly, all particles including the smallest (red) particles were retained with 96% of the maximum available voltage (Fig. 7a). Another experiment with only blue and orange particles was also carried out. The blue particles got caught in the hairpin while orange particles continued with the flow (Fig. 7b).

**Figure 8:**
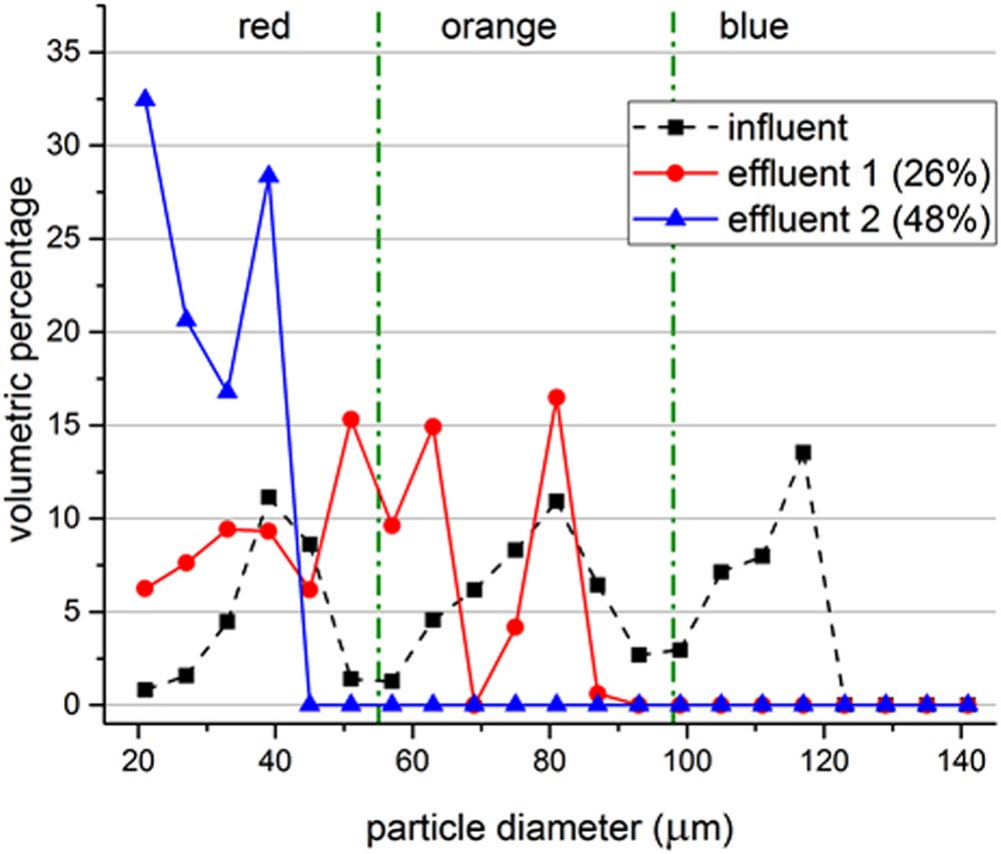
Particle size distributions before and after treatment. Measurement data of the original mixture (influent), treatment with 26% of the maximum available voltage (effluent 1) and treatment with 48% of the maximum available voltage (effluent 2) is shown with markers. Green dash-dot lines represent the approximate cut-off sizes between different colors of particles. Lines are used to guide the eye. Flow rate of the system is set to 100 mL h^−1^ while the excitation frequency is 1924 kHz. Data represents the average of 3 samples, where each sample is measured 3 times by DIPA2000 with standard deviations between 7.25 µm and 28.68 µm, where the largest standard deviations were obtained for the influent.

After some time, multiple particles trapped in a single node will form particle aggregates. As the aggregate gets bigger its size gets comparable to the wavelength. The particles on the edge of the aggregate are no longer experiencing sufficient acoustic radiation force to counter the drag. Therefore, these particles got detached from the aggregate and got trapped by a nearby node. Although the experiments were not continued long enough to explore the limits, eventually the nodes will be saturated and the set-up will require a washout. The prototype was kept in a horizontal position during the experiments and the aggregates did settle down as they grew bigger. Also, some particles got stuck in the grooves of the 3D printed part. The stuck particles can be removed by a washout. Figure 8 illustrates the volume histogram of the original mixture, treatment with 26% of the maximum available voltage and treatment with 48% of the maximum available voltage. After the treatment with 96% of the maximum available voltage, particle size analyzer was unable to detect particles in the given range.

Assuming the resistance of the system is independent of the applied voltage, the power consumption scales with voltage squared. Thus, 96% of the maximum voltage input approximately corresponds to 92% of the maximum power input. Other power input ratios can be calculated similarly.

**Figure 9:**
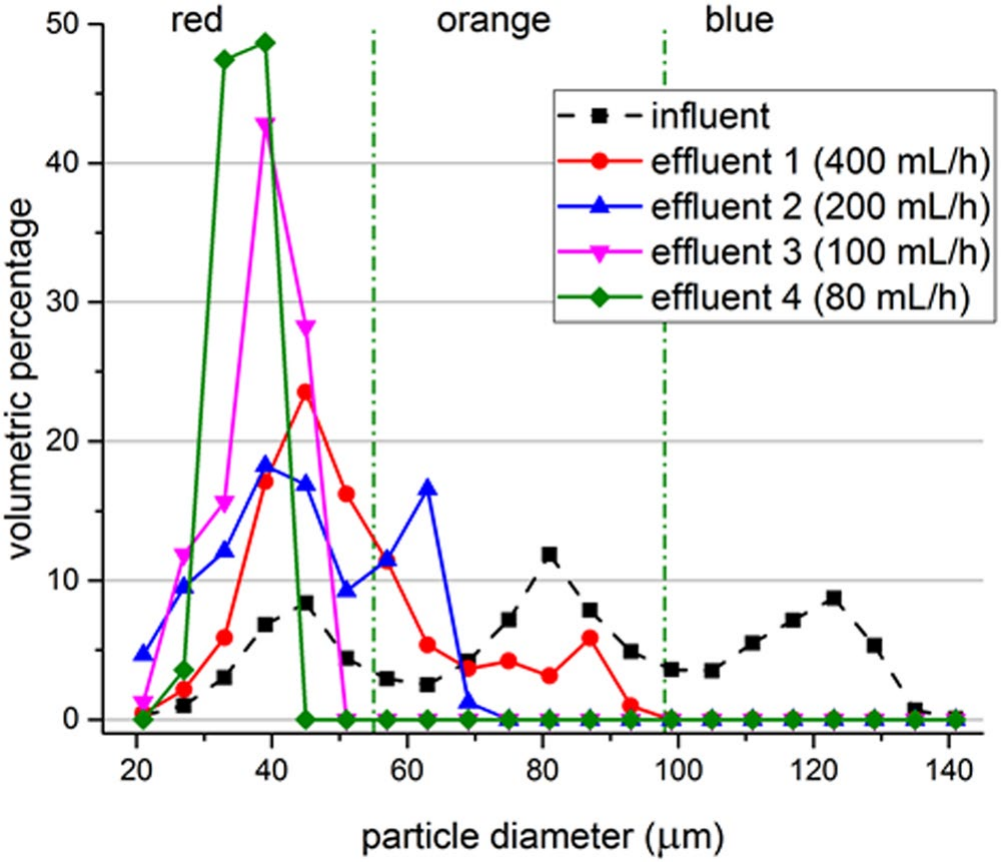
Particle size distributions before and after treatment. Measurement data of the original mixture (influent), treatment with 400 mL h^−1^ (effluent 1), treatment with flowrate of 200 mL h^−1^ (effluent 2), treatment with 100 mL h^−1^ (effluent 3) and treatment with 80 ml h^−1^ (effluent 4) is shown with markers. Green dash-dot lines represent the approximate cut-off sizes between different colors of particles. Lines are used to guide the eye. Voltage input to the system is set to 50% while the excitation frequency is 1924 kHz. Data represents the average of 3 samples, where each sample is measured 3 times by DIPA2000 with standard deviations between 3.39 µm and 29.87 µm, where the largest standard deviations were obtained for the influent.

The selectivity can also be achieved by altering the flow rate while keeping the voltage input constant for mixture (1). Voltage input was set to 50% of the maximum available voltage and the flow rate is changed step-wise. Figure 9 illustrates the volume particle size distribution histogram of the original mixture, treatment with 400 mL h^−1^, 200 mL h^−1^, 100 mL h^−1^ and 80 ml h^−1^ of input flow rate. Thus, the separation threshold can be shifted to smaller sizes by either increasing the voltage input or decreasing the input flow rate. It can be shifted to a larger size by doing the opposite.

**Figure 10:**
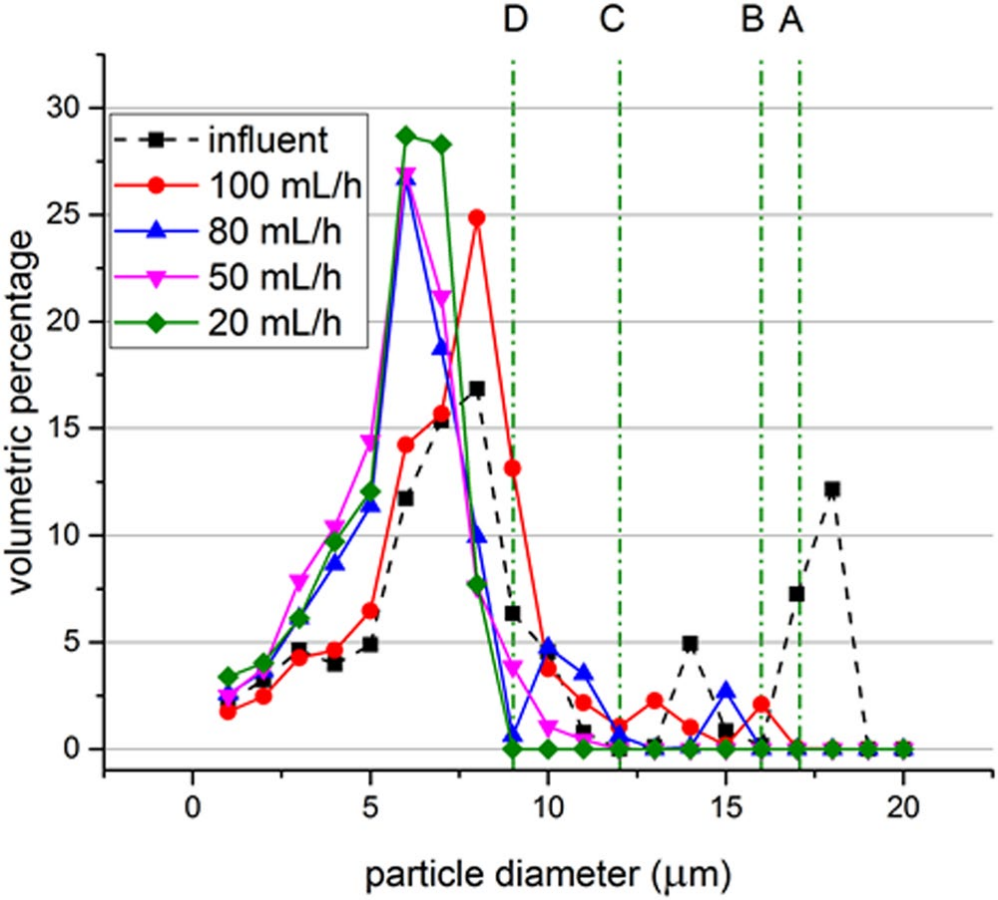
Particle size distributions for the untreated beer samples and after treatment with different flow rates while operating at 1972 kHz and the maximum available voltage. Lines A, B, C and D represent the separation thresholds for 100 mL h^−1^, 80 mL h^−1^, 50 mL h^−1^ and 20 mL h^−1^, respectively.

While processing mixture (2), the wheat beer, the acoustic separator was operated at 1972 kHz and at the maximum voltage as the size of the particles are small and acoustic contrast factor is unknown. Flow rate in the syringe pump was adjusted to 100 mL h^−1^, 80 mL h^−1^, 50 mL h^−1^ and 20 mL h^−1^, respectively. Figure 10 presents the volume histogram of samples taken before and after treatment with different flow rates. The untreated sample contained yeast up to 19 μm diameter. Treatment with 100 mL h^−1^ filtered the particles larger than 17 μm and with 20 mL h^−1^ flow rate particles larger than 9 μm were filtered.

Experiments and simulations confirm that the particles are trapped at the hairpin sections. Acoustic forces are acting in the vertical direction and the flow speed in vertical direction is lower in the hairpin sections. The acoustic force experienced by particles is large enough to counter the drag force around the hairpins. Ideally the acoustic field is a plane acoustic wave field and therefore the peak acoustic pressure is similar throughout the field. If the acoustic force is large enough to trap a particle in the middle of the channel, it can also trap the particle at the hairpins due to a locally lower velocity in vertical direction (Fig. 10). The aim of trapping larger particles selectively in the middle section may result in trapping smaller particles around the hairpins and in turn no selective separation or less efficient separation takes place. Thus, selective separation is achieved around the hairpins while the straight middle sections will carry remaining particles to the next hairpin.

This study demonstrated the possibility for selective filtering in an acoustophoretic serpentine channel. Interplay of the acoustic and drag forces results in particle trapping in hairpin sections, and multiple hairpin sections provide multiple areas for particles to be retained. The acoustic field inside the prototype may be improved by changing the inside wall material with a material that provides stronger reflections than PLA. Ensuring that the flow will be laminar and fully developed before entering the hairpins, shortening the linear sections and therefore the distance between the source and reflector would also provide stronger acoustic field. With a stronger acoustic field, the separator can be operated more efficiently and with higher flow rates.

## Conclusions

A standing wave acoustic field is generated by a piezoelectric transducer and the resulting acoustic radiation force is able to selectively trap particles in the hairpin sections of the serpentine separator. Simulations confirm that particles are captured in the hairpins of the separator. A single hairpin does not capture all targeted particles, but as the system contains multiple hairpins, effective particle trapping is ensured. Selective separation is achieved by changing the voltage input or flow rate in the case of model polyethylene particles. For wheat beer, the separation threshold is tuned by changing the input flow rate only. Both types of experiments demonstrated clear separation thresholds. Decreasing the flow rate or increasing the power input moves the threshold towards smaller particle sizes while increasing the flow rate or decreasing the power input creates the opposite effect. The efficiency of the system can be improved by adjusting the channel dimensions and reducing the losses in the system; stronger acoustic field will result with higher throughput possibility.
